# Rapid evolution of a Batesian mimicry trait in a butterfly responding to arrival of a new model

**DOI:** 10.1038/s41598-017-06376-9

**Published:** 2017-07-25

**Authors:** Mitsuho Katoh, Haruki Tatsuta, Kazuki Tsuji

**Affiliations:** 1 Department of Agro-Environmental Sciences, Faculty of Agriculture, University of the Ryukyus, Okinawa, 903-0213 Japan; 2 The United Graduate School of Agricultural Sciences, Kagoshima University, Korimoto 1-21-24, Kagoshima, 890-8580 Japan

## Abstract

Batesian mimicry, a phenomenon in which harmless organisms resemble harmful or unpalatable species, has been extensively studied in evolutionary biology. Model species may differ from population to population of a single mimetic species, so different predation pressures might have driven micro-evolution towards better mimicry among regions. However, there is scant direct evidence of micro-evolutionary change over time in mimicry traits. *Papilio polytes* shows female-limited Batesian mimicry. On Okinawa, one mimicry model is *Pachliopta aristolochiae*, which was not present on the island until 1993. In *P*. *polytes*, the size of the hind-wing white spot, a mimetic trait, is maternally heritable. Among specimens collected between 1961 and 2016, the average white spot size was unchanged before the model’s arrival but has rapidly increased since then. However, white spot size showed greater variance after the model’s establishment than before. This suggests that before 1993, white spot size in this population was not selectively neutral but was an adaptive trait for mimicking an unpalatable native, *Byasa alcinous*, which looks like *P*. *aristolochiae* apart from the latter’s hind-wing white spot. Thus, some females switched their model to the new one after its arrival.

## Introduction

Recent studies in evolutionary biology have demonstrated that micro-evolutionary change is directly observable in the field when natural selection is strong^1^, with some remarkable examples of the rapid evolution of morphological traits^2, 3^. Batesian mimicry, whereby organisms without a capture cost resemble species that bear a capture cost^4^, has been regarded as strong indirect empirical evidence of the past operation of natural selection^4–8^. However, there is scant direct evidence of real-time micro-evolutionary change in mimicry traits except for the coral snake – king snake case, in which rapid evolution of better Batesian mimicry was observed when the model’s density became extremely low^9^. Here, we describe an example of rapid evolution of wing coloration pattern, a Batesian mimicry trait, in a butterfly.

**Figure 1:**
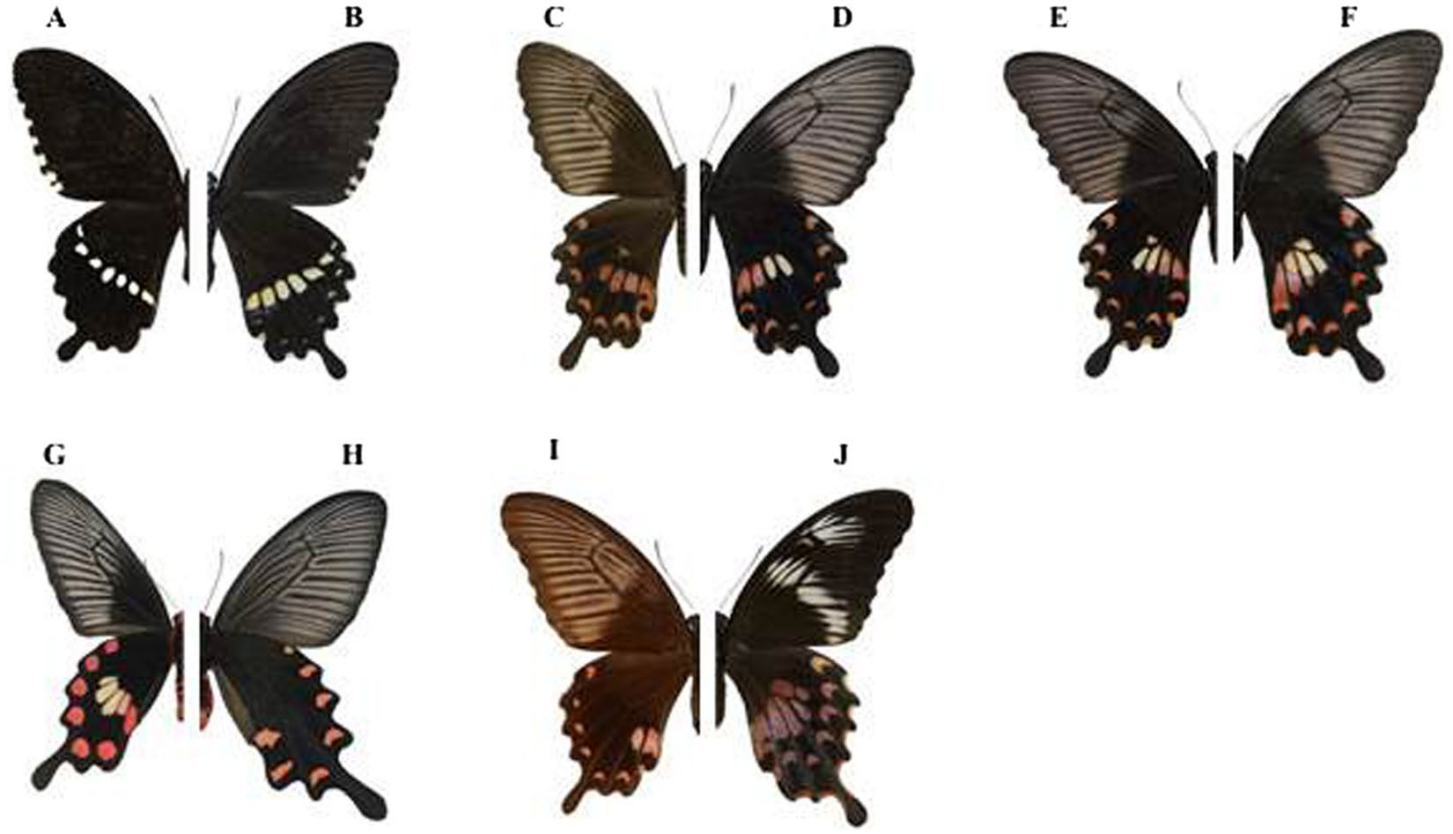
(**A**) A *Papilio polytes* male and (**B**) a *P*. *polytes* f. *cyrus* non-mimetic female, with basically the same wing colour pattern. (**C**–**F**) *P*. *polytes* f. *polytes* resembles (**G**) *Pachliopta aristolochiae*. As shown, however, there is a large variation in wing patterns among f. *polytes* females. Some females, for example (**C**), lack a white spot in the hind wing, resembling (**H**) *Byasa alcinous*, another unpalatable butterfly. (**I**) *P*. *polytes* f. *theseus* and (**J**) f. *romulus* (from the University Museum, University of Tokyo), which are not distributed in Japan, are considered to mimic ‘red plain models’.

The swallowtail butterfly, *Papilio polytes*, is a common species throughout South-East Asia. This species has a female-limited polymorphism in Batesian mimicry, in which females exhibit four morphs (forma *cyrus*, f. *polytes*, f. *romulus* and f. *theseus*), whereas males are always monomorphic within a population^6, 10^. Females of each morph of *P*. *polytes* are morphologically differentiated in closely resembling sympatric unpalatable butterflies^6^. Forma *cyrus* (Fig. 1B) is the non-mimetic morph that resembles males of the same species (Fig. 1A), with a line of white spots on the black hind wings. The other three female morphs are considered to mimic other unpalatable species of the Papilionidae, allowing them to avoid predators^7, 11^. Single-locus Mendelian inheritance coded by the autosomal *doublesex* supergene, with mimetic alleles dominant to the non-mimetic allele, controls this polymorphism^10, 12^. The geographic distribution of the mimetic f. *romulus* (Fig. 1J) and f. *theseus* (Fig. 1I) is limited to the true tropics, where they share their range with ‘red plain models’ such as *Pachliopta aristolochiae* (black form) and *Pachliopta hector* ^6^. Their wing morphology is characterized by many small red spots on the black hind wings. In contrast, f. *polytes* (Fig. 1C–F), with a broad distribution including subtropical China and Japan, is considered to mimic ‘white spot models’, including *P*. *aristolochiae* (white form, Fig. 1G), *Pachliopta polyphontes* and *Pachliopta polydorus* ^6^, whose distributions roughly overlap that of f. *polytes*. Forma *polytes* and its models are characterized by a conspicuous white spot in the centre of the hind wing surrounded by several small red spots (Fig. 1D–F). These various wing colour patterns suggest that regionally different predation pressures related to differences in model morphology drove micro-evolution towards better mimicry in each region.

The Ryukyu Archipelago, situated in the subtropical region of Japan, lies at the northern end of the species distribution of *P*. *polytes*. Forma *polytes* is the only mimetic morph found on the Ryukyus. Historically, its putative ‘white spot model’ was not distributed in Japan. The distribution of *P*. *aristolochiae* has been expanding recently, however, and this species began establishing populations on some islands of the Ryukyus in the 1960s. Uesugi^13^ observed a rapid increase in the proportion of mimetic females after the arrival of the model on Miyako Island, in what can be regarded as a direct observation of the evolutionary process of Batesian mimicry^13, 14^. Uesugi^13^ also noted that the size of the hind-wing white spot of mimetic females was highly variable in the Ryukyus, and that the average spot size was larger on islands where the model *P*. *aristolochiae* was distributed than on islands where it was absent, including Okinawa (the main island of the Ryukyus). Two years after Uesugi’s report, in 1993, *P*. *aristolochiae* established a population on Okinawa^15^ (the distribution record of Clarke and Sheppard^3^ has a mistake: *P*. *aristolochiae* was not distributed on Okinawa in 1972). This provides us with another fortunate opportunity to directly observe the presumable micro-evolutionary change in the mimetic wing colour patterns.

We hypothesized that because of the changed predation pressure due to the immigration of the white spot model, natural selection (directional and stabilizing selection) would have driven the wing colour patterns of f. *polytes* to more closely resemble the model from the former selectively neutral state in the absence of the model. To test this hypothesis, we first estimated the heritability levels of the red and white spot patterns of f. *polytes* in the field population. The presence of heritable variation in those quantitative traits enables micro-evolution in response to natural selection^16^. Second, using specimens in museum and private collections and some captured by us, we analysed quantitative changes in the size of the white spot over more than five decades in the field populations of Okinawa and other islands (the Miyako and Yaeyama groups) of the Ryukyus.

## Results

### Estimation of heritability of mimicry traits

The broad-sense heritability (*H* ^2^) of the white spot size (relative to the hind-wing area) was significant, whereas that of the red spot size was not significant (Table 1). The white spot size was inherited only maternally, with heritability exceeding 1 (Table 1). There was no significant genetic correlation between the white and red spot sizes (maternal *r* = 0.152, *P* = 0.134; paternal *r* = 0.065, *P* = 0.351). These results indicate that the white spot size in the mimetic females can change in response to natural selection.

**Table 1:** Estimates of heritability of the mimetic spot size of *P*. *polytes* f. *polytes* derived from grandmother–granddaughter regression.

Colour of spot	Heritability	CI (2.5:97.5)	*P*
White			
Maternally (broad sense, *H* ^2^)	2.40	1.268:4.628	0.013
Paternally (narrow sense, *h* ^2^)	1.12	−1.104:4.208	0.292
Red			
Maternally (broad sense, *H* ^2^)	0.75	−1.700:2.160	0.482
Paternally (narrow sense, *h* ^2^)	−1.50	−3.572:−0.044	0.151

*P*-value indicates significance of the regression.

### Change in spot size over time

**Figure 2:**
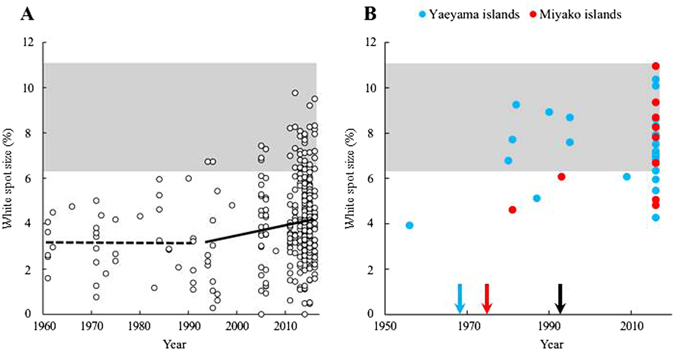
(**A**) Change in the white spot size (as a percentage of hind-wing area) of *P*. *polytes* f. *polytes* over the last 55 years on Okinawa. The dashed line is the regression of the white spot size before the new model’s settlement (1961–1991); the solid line is the regression after the new model’s settlement (1994–2016). The grey band represents the range of the mean ± 2SD of the model, *P*. *aristolochiae*. (**B**) Change in the white spot size of f. *polytes* in the Miyako group (red dot: including Miyako and Irabu islands) and the Yaeyama group (blue dot: Ishigaki, Hateruma and Iriomote islands). The grey band is the range of the white spot size of the model. The arrows show the years when *P*. *aristolochiae* established populations on each group.

Using specimens in museum and private collections and some captured by us, we analysed changes in the size of the white spot over the last 55 years in the field population on Okinawa. The results showed that the white spot size increased during that period (Fig. 2). It did not change between 1961 and 1991, when the model was absent (regression coefficient *b* = −0.005, *P* = 0.819), but it increased from 1994 to 2016, after the model’s arrival in 1993 (*b* = 0.045, *P* = 0.031). Over time, the white spot size approached the model’s range (Fig. 2A). The variance of the white spot size was greater after 1993 than before (*F* = 0.56, df = 35, 331, *P* = 0.044, Fig. 2A).

In the Yaeyama and Miyako island groups, where the model arrived earlier (in 1968 and 1975, respectively), the white spot size seemed to increased over the last 61 years (1955–2016, *b* = 0.0284, *P* = 0.145). Remarkably, the timing seemed to differ from that on Okinawa, with a more prominent increase in size before 1992 (*b* = 0.111, *P* = 0.172) than after 1993 (*b* = 0.006, *P* = 0.885), although neither was significant (Fig. 2B). During the 1980s, the white spot size was larger in the Yaeyama and Miyako island groups than on Okinawa (Student’s *t*-test, *t* = 3.79, *P* < 0.01). The spot size of f. *polytes* in the Yaeyamas and the Miyakos now (in 2016) greatly overlaps with that of the model (Fig. 2B).

## Discussion

Batesian mimicry has been regarded as a textbook case for natural selection theory^5, 7^. Our results confirm this assertion with real-time micro-evolution data for the following reasons. We clearly showed that the white spot size of *Papilio polytes* f. *polytes* on Okinawa changed rapidly to better mimic the model *Pachliopta aristolochiae* after the model’s arrival. A similar change occurred earlier on the Miyako and Yaeyama islands, where the model arrived earlier. These results strongly suggest that the observed change in wing coloration of f. *polytes* was caused by the arrival of the model, not by an environmental change. Furthermore, there is experimental evidence that birds on Miyako Island that fed on *P*. *aristolochiae* started to avoid *P*. *polytes* f. *polytes* ^13^. Thus, f. *polytes* in the Ryukyus really mimics *P*. *aristolochiae*.

However, the increased variance of the white spot size after 1993 might contradict our above scenario, because we predicted reduced variance after the model’s establishment, owing to presumable stabilizing selection favouring better mimicry. One explanation is that it is an artefact due to the small sample size before 1992: we might have failed to sample outliers before the model’s arrival just by chance. However, we consider this to be unlikely, because the white spot size variation showed a good fit to a normal distribution, where outliers might be less important. Another explanation is that the mimetic trait of *P*. *polytes* on Okinawa before the arrival of *P*. *aristolochiae* was not a neutral trait but was an adaptive trait mimicking another model. In 1991, Uesugi^17^ assumed that the model of f. *polytes* on Okinawa was the unpalatable butterfly *Byasa alcinous*, a native to the island. The wing colour pattern of *P*. *aristolochiae* (Fig. 1G) closely resembles that of *B*. *alcinous* (Fig. 1H) if the hind-wing white spot of *P*. *aristolochiae* is ignored. This explanation is consistent with the fact that before the arrival of *P*. *aristolochiae*, mimetic females of *P*. *polytes* had smaller white spots on average (Fig. 2). Thus, some of the mimetic females might have switched their model to *P*. *aristolochiae*. The increased variance of the white spot size of mimetic *P*. *polytes* after the arrival of *P*. *aristolochiae* can be explained by disruptive selection due to the presence of two models, one with and the other without a white spot.

This new scenario still leaves one question, namely why most mimetic females before the arrival of *P*. *aristolochiae* still had the white spot, even if it was smaller. In other words, why did they not exhibit a phenotype like another mimetic form found in the tropics, f. *theseus*, which totally lacks the white spot? To answer this question, we need to ascertain how long *P*. *polytes* populations have inhabited Okinawa. Furthermore, studies are needed to elucidate what genetic and developmental changes underlie the micro-evolution described above. The female-limited Batesian mimicry in *P*. *polytes* is coded by the autosomal *doublesex* supergene^10, 12^. However, our analysis indicates that the white spot size is only maternally inherited, and thus the white spot size variation in the studied population is not controlled by an autosomal gene. So a gene that controls white spot size may exist on the W sex chromosome (lepidopterans have ZW sex determination, in which females are heterozygous) or in organelle genomes. The result that the estimated heritability of the white spot size is >1 supports this idea. The grandmother–granddaughter regression model we used assumes autosomal gene control. The alternative assumption of W chromosome or organelle gene control would make the true heritability 1/4 of the values shown in the Results.

This study provides direct evidence of rapid micro-evolution of a mimetic trait in response to community changes due to the extension of the distribution of a new model. This is the first study to directly observe the theoretical process of the evolution of Batesian mimicry, that is, local adaptation to a mimetic trait to better resemble a new local model.

## Methods

### Quantification of mimicry traits

**Figure 3:**
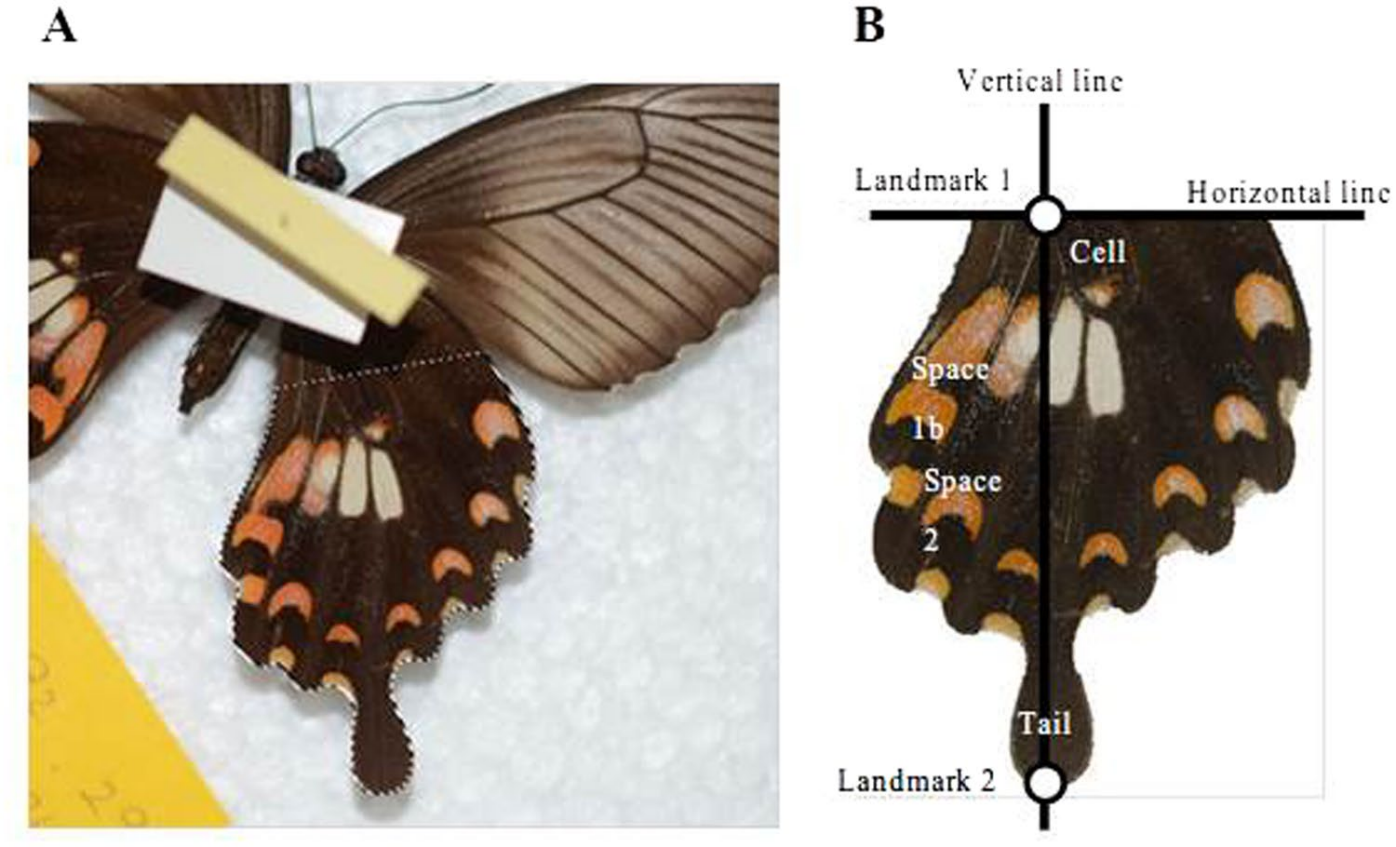
(**A**) A butterfly specimen from the University Museum, University of Tokyo. The hind-wing area bounded by a dotted line was used for the analysis. (**B**) Image trimming.

A wing of each butterfly (ventral view) was photographed with a digital camera (Nikon D7000, Nikon Corporation, Tokyo, Japan) mounted 50 cm directly above the specimen under invariant light (Fig. 3A). The left wing was used unless it was damaged. We analysed the hind-wing area below a horizontal line (Fig. 3B) demarcated as follows. We defined landmark 1 as the intersection of the second anterior cubitus vein (the border between space 1b and space 2) and the border of the cell on the hind wing, and landmark 2 as the tip of the vein on the tail of the hind wing. The vertical line connecting landmarks 1 and 2 was rotated so that it was vertical in Adobe Photoshop CS5 (Adobe Systems Inc., San Jose, CA, USA). Then a horizontal line crossing landmark 1 was drawn perpendicular to the vertical line. The white (or red) spot size was defined as the sum of the total area of the white (or red) spots on a hind wing divided by the total hind-wing area (the area bounded by a dotted line in Fig. 3A) measured in ImageJ^18^ image analysis software. In the majority of specimens (*N* = 347), the white spot was distinct from the background black area and from red areas, and was easily identified in the image. However, in some specimens (*N* = 3 before 1993, *N* = 18 after 1993), such distinction was unclear, because adjoining white and red spots were partially fused. In such cases, we chose 30 points from the clearly white spot area at random with the ‘multi-point’ tool of ImageJ, converted the image to grey scale, and evaluated the brightness value (0–255) of each pixel. We defined a pixel as being included in the white spot area when its brightness value was higher than the mean − SD brightness value of the focal butterfly. When this procedure was tested on the specimens with distinct white spots, the number of pixels judged as white spot area was almost exactly the same as that identified visually. We first analysed red and white spot sizes separately, and then analysed their genetic correlation.

We similarly measured the spot sizes of the model, *P*. *aristolochiae*, for comparison.

### Estimation of heritability of mimicry traits

Adult females (*N* = 16) of *P*. *polytes* f. *polytes* were collected in Nakijin village, Okinawa Island, in 2011. Offspring of those females were reared in the laboratory (25 ± 1 °C, L/D 12:12) on fresh *Toddalia asiatica* leaves for three consecutive generations. Breeding was random on the condition that mated pairs shared neither parents nor grandparents in order to avoid inbreeding. Each adult was allowed to mate only once. Because the mimicry is a female-limited trait, we used grandmother–granddaughter regression to estimate the heritability of spot size, separately analysing paternal grandmother–granddaughter (for estimation of narrow-sense *h* ^2^) and maternal grandmother–granddaughter (for estimation of broad-sense *H* ^2^) regressions^19^. To estimate heritability from the phenotypic similarity between second-degree relatives, the correlation coefficient among grandmother–granddaughter pairs, *r* (with a confidence interval estimated by bootstrapping), is quadrupled: *h* ^2^ (or *H* ^2^) = 4*r* ^19^. The 95% confidence intervals (CIs) of the heritabilities were estimated by the bootstrap method, using 10 000 repetitions for each parameter set. Genetic correlation between white and red spot sizes is also estimated similarly to the above, except for the correlation was that between grandmother red (white) spot and granddaughter white (red) spot.

### Change in spot size over time

We searched in museums and private collections for specimens of the mimetic form of *P*. *polytes* collected during the last 55 years (1961–2016) on Okinawa, and in 2011–2016 collected specimens ourselves as well (Table 2). After phenotypic measurements, we analysed changes in the mimetic trait over time. To compare the trends before and after the new model’s arrival, we performed least squares linear regression analysis for 1961–1991 (before the new model’s arrival) and 1994–2016 (after). We also searched museum and private collections for the mimetic form captured on other islands in the Ryukyus, namely the Yaeyama group (where the model arrived in 1968) and the Miyako group (where it arrived in 1975)^17^. The small sample size before the 1980s hindered comparison before and after the model’s settlement. So we combined the data from the Yaeyamas and the Miyakos for linear regressions of white spot size against year, using data sets before and after 1993. We predicted that white spot size has also changed on those islands but at a different time from that on Okinawa Island.

**Table 2:** Specimen information. *Denoted simply as ‘Okinawa Island’ when the locality on the island was not identified on the label.

Collection holder	Place of collection	Year of collection	Sample size	ID of specimen
University Museum, Univ. of Tokyo	Ishigaki Island	1956	1	1192, 38
University Museum, Univ. of Tokyo	Okinawa Island*	1962	1	1192, 35
University Museum, Univ. of Tokyo	Okinawa Island	1966	1	1192, 36
National Museum of Nature and Science	Okinawa Island	1961	5	
National Museum of Nature and Science	Okinawa Island	1962	1	
Okinawa Pref. Museum and Art Museum	Nanjo city	1971	1	89
Okinawa Pref. Museum and Art Museum	Nanjo city	1972	1	90
Okinawa Pref. Museum and Art Museum	Naha city	1973	1	123
Okinawa Pref. Museum and Art Museum	Hateruma Island	1980	1	
Okinawa Pref. Museum and Art Museum	Irabu Island	1981	1	
Ryukyu University Museum (Fujukan)	Okinawa Island	1971	7	
Ryukyu University Museum (Fujukan)	Nishihara city	2008	1	
Ryukyu University Museum (Fujukan)	Iriomote Island	2009	1	
Kazuki Tsuji	Motobu city	1975	1	
Kazuki Tsuji	Nanjo city	1980	1	
Kazuki Tsuji	Iriomote Island	1981	1	
Kazuki Tsuji	Ishigaki Island	1982	1	
Kazuki Tsuji	Nakijin village	1991	2	
Kazuki Tsuji	Nanjo city	1991	3	
Kurashiki Museum of Natural History	Gushiken village	1975	2	
Kurashiki Museum of Natural History	Miyako Island	1993	1	
Nago Museum	Nago city	1983	1	1112
Nago Museum	Motobu city	1984	1	1109
Nago Museum	Motobu city	1984	1	1129
Nago Museum	Nishihara city	1999	1	
Itami City Museum of Insects	Nanjo city	1984	1	20–15
Itami City Museum of Insects	Gushiken village	1984	1	20–13
Itami City Museum of Insects	Ishigaki Island	1987	1	26. VIII
Itami City Museum of Insects	Nanjo city	1988	1	20–11
Itami City Museum of Insects	Ishigaki Island	1990	1	23. IX
Itami City Museum of Insects	Nanjo city	1990	1	20–14
Okinawa Municipal Museum	Okinawa city	1986	2	
Okinawa Municipal Museum	Motobu city	1994	4	
Nat. Inst. for Agro-Environmental Sci.	Ishigaki Island	1995	2	
Senshi Nobayashi	Motobu city	1995	1	
Senshi Nobayashi	Nakijin village	1995	5	
Senshi Nobayashi	Motobu city	1996	2	
Senshi Nobayashi	Nakijin village	1996	1	
Toshiki Hatano	Okinawa Island	2005	18	
Toshiki Hatano	Okinawa Island	2006	15	
Mitsuho Katoh	Nakijin village	2011	20	
Mitsuho Katoh	Nakijin village	2012	20	
Mitsuho Katoh	Nakijin village	2013	26	
Mitsuho Katoh	Nakijin village	2014	73	
Mitsuho Katoh	Nakijin village	2015	110	
Mitsuho Katoh	Ishigaki Island	2016	15	
Mitsuho Katoh	Miyako Island	2016	8	
Mitsuho Katoh	Nakijin village	2016	35	

### Statistical analyses

We used R v. 3.2.3 software^20^ for all statistical analyses. We first tested the fit of the white and red spot sizes to a normal distribution by the one-sample Kolmogorov–Smirnov test (in the *ks*.*test* package), separately testing the field-collected samples (temporal change analysis for the samples from Okinawa and other islands separately) and the laboratory-reared samples (heritability estimation). In all samples, neither distribution deviated significantly from normal. In all subsequent analyses we used general linear models assuming a normal distribution. We calculated the correlation of spot size between granddaughters and grandmothers for the estimation of heritability and CI as explained above. We performed a least squares linear regression of white spot size against year to detect any change over time. The significance of correlation and regression coefficients was tested using *F* statistics. Variance in white spot size on Okinawa between before and after arrival was compared by *F*-test.
